# Successful Case of Ovariatomy

**Published:** 1860-08

**Authors:** Wm. H. Byford

**Affiliations:** Prof. of Obstetrics, &c., in Medical Department of Lind University


### SUCCESSFUL CASE OF OVARIATOMY.

By Wm. H. BYFORD, A. M., M. D.
Prof. of Obstetrics, &c., in Medical Department of Lind University.

Miss E., aged 15 years, from Roseville, Ill., was brought to me by her parents, May 15th, 1860, to consult me about an abdominal enlargement of which she was the subject. Upon examination, I found a multilocular ovarian tumor of very large size. It occupied the whole abdominal and pelvic cavities, and produced an enlargement much greater than pregnancy at full term.

The history of the case was very obscure, and all that could be obtained was furnished me by Dr. John A. Young, of Monmouth, who was her physician. Dr. Y. says: "Her mother informs me that she was always peculiarly shaped, being short waisted, and as she expressed it, her sides very full and hard; abdomen full. She menstruated at 13, I believe, and continued regular until sometime in the winter, when she missed two or three periods in succession. About that time they noticed sensible increase in size, when they called upon me for advice. She would not submit to any examination, save a very imperfect one of the abdomen externally. There was evident fluctuation, and I noticed in the right iliac region some apparently solid body, about the size of a large orange. I was unable to elicit from her, or from her mother, any reliable history of her enlargement; or whether they had ever noticed any tumors of any size, at any point before. I put her upon the use of Hyd. Potas., with Spts. Nit. and Buchu. Since then, her father informs me, that the catamenia have returned."

After satisfying myself as to the nature of the tumor, I informed them that there were two modes of operative proceedure usually resorted to in such cases, viz: Tapping, and iodine injections, and extirpation. That the former probably afforded her not five per cent. of the chances of recovery, on account of its frequent failure to obliterate the sacs, and that this was a particularly unfavorable case, as there were at least seven cists, all of which would require the puncture and injection.

That the latter operation never failed to eradicate the disease, but was most hazardous, from peritoneal inflammation and other consequences, which in the nature of it must be risked; if such effects were not fatal, the cure would be perfect. That I believed with the operation of extirpation the chances would be seventy-five per cent. in her favor. I felt justified in making this statement from the great perfection to which this operation has been recently brought by the labors of English Surgeons, (and especially Mr. Spencer Wells,) the excellent health of the patient, and the strong conviction that there were no peritoneal adhesions.

After consultation among themselves they chose ovariatomy, and begged me to appoint a time when I would attend to it. As her menses were close at hand, it was deemed best to postpone the operation until the 30th of May.

At this last date I proceeded to operate, assisted by Drs. Young, Hamilton, and McDill, of Monmouth, and Taliaferro, of Roseville; in the following manner:

After having the room brought up to 98 degrees Fahrenheit, the patient was fully etherized and placed upon her back on the operating table; an incision through the abdominal walls, about midway between the umbilicus and symphisis pubis in the linea alba, three and a half or four inches long, exposed the tumor. This being done, I plunged a large trocar into it, and emptied one of the cists of probably two pounds of thick albuminous transparent fluid. Soon as it ceased to run I withdrew the trocar, and introduced it into another sac, and so on one after another until eight cists were evacuated, one of which contained eighteen pint cup-fulls of liquid. The abdomen was now very much collapsed, and the tumor so reduced in size that it was easily withdrawn through the incision. As it lay upon the table, the peduncle, which was about two inches broad, passed through the wound. Two strong silk ligatures were passed, one through each edge of the peduncle, near the tumor, in order to give us command of the stump after amputation. The ecrasseur was passed around the peduncle, between the ligatures and the ovary, and with it the mass was separated from its attachment. The stump was drawn between the lips of the incision, near its lower angle, and held in position by the two ligatures. Then silver pins, with steel points, were passed through the lips of the wound, passing near to, but not quite touching the peritoneum, and secured with thread as in hare lip. A few fine silver wire sutures closed up the wound superficially. Two of the silver pins used in transfixing the lips of the wound also passed through the stump of the detached tumor, and held its amputated margin a little above the level of the skin of the abdomen. A compress wet with cold water was placed upon the wound, all surrounded with a flannel binder, and the patient placed carefully back in bed. In about an hour she awoke from her sleep perfectly conscious, but entirely ignorant of what had occurred with respect to the operation, and expressed herself as perfectly comfortable.

The cists and contents weighed thirty-nine pounds, and as it was an ordinary many cisted mass it would be unnecessary to give a particular description of it.

As the case was some two hundred miles distant from Chicago, I will introduce the notes of Dr. Taliaferro, as they were communicated to me in letters from Dr. Young.

These two gentlemen had the case under their care after the operation, and much is due to their assiduous and skillful attention for the successful issue to which it was conducted.

The following are Dr. Young's letters:

Monmouth, May 31st, 1860.

Dr. Byford,

Dear Sir:

I visited Mr. Eldridge's this morning at 9 o'clock; the patient reported favorably. I will first transcribe Dr. Taliaferro's memorandum.

May, 30th, 2 o'clock, p. m.—$\frac{1}{8}$ gr. Morphia: at 3, dozing; pulse 112. 4 p. m.—$\frac{1}{8}$ gr. Morphia. 5.30—Slept comfortably last half-hour; pulse 100. 7.30—Comfortable, not slept any since 6. Evacuated bladder naturally; pulse 108.

31st, 6 a. m.—Since last note, 1 gr. Opium every two hours. Expressed herself as feeling comfortable; some dryness of the mouth, but little thirst. Passed urine this morning naturally.

I arrived about 9 a. m.—found her resting comfortably; she says she has no pain or soreness of any part; has slept pleasantly and feels refreshed. Skin moist; pulse 104, soft and good volume. I interrogated her with regard to the feeling of the wound, as I had concluded not to disturb the the dressing unless there was some sufficient cause. She said all felt well; there was no pain, heat, or soreness, and no fulness or tight sensation. I therefore concluded there was nothing more to be done at present. About 12 o'clock, when I was making preparation to leave, Mrs. Eldridge asked me if it would be safe to remove the wet things from about her; upon enquiry as to what *wet things* she alluded, my suspicions became aroused, and I immediately examined the binder and compresses, when I found them thoroughly wet with blood, and a large clot lying immediately under and around them. Being surprised at the apparent amount of loss, I examined the pulse again, for fear that I might have been mistaken, but found it as before, 104, regular and good volume. I sponged off the wound, and found that the hemorrhage had proceeded from the pedicle. Kept it exposed some time and watched it closely, there was no return of it. I covered that portion of it from which the bleeding had occurred with Pulv. Sulph. Alum, and small dossils of lint. We then moved her sufficiently to change all the wet things from about her. Examined again, no return of bleeding. Applied *compress*—lighter than before —with binder, and directed Dr. T. to watch closely for a return, and should it recur, to try and secure by ligature if possible. When I left, about 1.30, she said she felt very well. I hope there will be no return of it, yet I fear it. I very much wish we had applied the double ligature in connection with the ecrasseur. The hemorrhage was all external as far as I could determine.

---

Monmouth, June 1st, 1860.

Dear Dr.:

I am happy to inform you in advance that my report of to-day is more favorable and encouraging than that of yesterday. I commence, as before, with the notes of Dr. T.

May 31st, 3, p. m.—Quite comfortable, but little thirst, no pain, pulse 104; has had pleasant and refreshing sleep. 9, p. m.—Same; voided urine about 6 o'clock, naturally and without any trouble; Opium, 1 gr., every two and a half hours. 12, m.—Condition same; no appearance of hemorrhage.

I forgot to mention yesterday that in dressing the wound I placed dossils of lint in the inguinal region, just under the edge of the binder, to give warning if it should be renewed. These could be examined at any time without disturbing the patient.

June 1st, 7 a. m.—No change; pulse 101. Skin moist; slight perspiration when sleeping. Has taken 2 or 3 table-spoonfuls of crackers in coffee. Says she has passed a good night.

I arrived at 10 a. m. Patient looked bright and cheerful; said she felt well, better than yesterday; that her sleep had been refreshing. No particular desire for food, like hunger, but that every thing tasted well, and she could eat. Pulse 106, rather harder than yesterday; skin moist; no pain, soreness, or disagreeable sensation of any kind; mouth feels a little dry, but no actual thirst, a little ice or mouthful of cold water satisfies. Examined, superficially, the dressing, and found it all as I had left it on yesterday.

2.15, p.m.—Rested quietly; pulse 112; skin moist. Cheerful; says she thinks she is going to get well. Removed binder, and compressed carefully; found all as I had left it yesterday; no return hemorrhage; bowels a little more full, not amounting, however, to swelling; no tenderness at least on slight pressure. To continue treatment.

The hemorrhage of night before last and yesterday must have been slow, as the apparent amount lost, if withdrawn at once, or in a very short time, would assuredly have produced alarming symptoms. When I first discovered it I know it *alarmed* me. I hope, however, that there will now be no return of it.

Finding her in such favorable condition, and having fallen behind in my business, I have concluded not to return until Sunday morning. This, at any rate, is the last that you could hear from me until Monday evening next.

I directed her to have a spoonful of beef-tea occasionally, and a little soaked cracker. I deemed this the more necessary in consideration of the hemorrhage.

---

Monmouth, June 3d, 1860.

Dear Dr.:

It now gives me unfeigned pleasure to report the favorable progress of our interesting patient, as I found all right to day, I may say as well as could possibly be. But to the notes of Dr. T.:

June 1st, 9 o'clock, p. m.—Patient expresses herself as feeling comfortable; a little more heat of surface this p. m. than usual; hands and feet particularly. Sponged with moderately cool water about mid-afternoon; at present, temperature natural; skin moist, pulse 112; no pain or tenderness of bowels, though somewhat more flatulant. Voided urine three times to day, last at 6 o'clock, p. m.

2d, 1.30 a.m.—Resting comfortably; skin moist, amounts to sweating when asleep; pulse 110. 6, a.m.—Rested well; pulse 110; prespiring; bowels not so tympanitic as yesterday; voided urine about 5 o'clock. Has been taking Carb. Soda, about two or three grains every hour or two, with favorable effect on flatulence. 5, p.m.—Pulse 106. No heat of skin; rather unpleasant from sweating while asleap; voided urine about 3, p.m. Has taken a little tea and cracker occasionally since yesterday, says it tastes well; but little thirst. Tightened the bandage as it had become quite loose. 9.30, p.m.—Pulse 104; otherwise as during the day. Voided urine about 7.

3d. 5, a.m.—Pulse 97; other symptoms as before. Bandage more loose; adjusted again. I arrived at 10 a.m.; found her looking well and quite cheerful; says she feels stronger. Tongue clean—or with only a slight white coat, as we would see from abstinence and sleeping so much. Pulse 107; skin moist, perhaps more than necessary. Has been taking some chicken broth, and likes it much. Her Aunt thinks her appetite is good, but that she will not confess it for fear they might offer her too much, as she is aware that she was restricted in diet.

11, a.m.—Examined the wound, removing all the lint, except that immediately adherent to the point from where hemorrhage had occurred; that was firm and dry, occupying about a thumb's breadth. Balance of wound looked well; closed by first intention, and pretty firm. Not a drop of pus seen; cleansed the parts and dressed lightly. No tenderness upon pressure or handling; only slight fulness, not more than there should be in normal condition. Lower portion of abdomen, from umbilicus down, not even rounded up yet. Her countenance shows some evidence of the loss of blood, and also perhaps the effect of the perspirations, in being considerably blanched; her lips, however, retain a good color.

I deemed it prudent under the circumstances to direct a slight increase of nourishment; a table-spoonful or two of chicken-broth or beef-tea every two or three hours, watching, of course, the effect closely. To continue the opiate still as needed.

I would here state—although it does not appear in the body of the notes—that 1 gr. of opium had been given regularly every two and a half or three hours, and has exerted a happy influence, producing composure and pleasant and refreshing sleep.

2, p. m.—Pulse 96. I account for the difference by her slight excitement when I first arrive.

---

Monmouth, June 5th, 1860.

Dear Dr.:

I still have the pleasure of reporting favorably; no untoward symptom having as yet arisen. As you will perceive by the enclosed, I have removed the pins, everything looking well. I now recur to the regular notes.

June 3d. 9 p.m.—Pulse 100; other symptoms as previously noted. Sweating continues; Aromat. Sulph. Acid, 3 or 4 drops every six hours. I neglected to state in my last that I had advised Dr. T. to use that remedy in case the sweating continued so profuse.

4th. 5 a. m.—Had a comfortable night; pulse 85; sweating not so profuse; appetite increasing a little. 2 p.m.—Pulse

92; otherwise same as morning. Sweating a little more. Elix. Vit., 5 gtts. 9 p.m.—Pulse 92; Sweating diminished; gentle moisture only when asleep.

June 5th.—I arrived at 11 a.m.; found her looking well and feeling stronger; says she feels well, no soreness or pain whatever. Pulse 88; appetite better; food tastes well, and more desire for it. Abdomen natural in rotundity; some barborygmus; bowels in evident motion, but without producing pain. Has taken no opium since 1 o'clock, p.m. Removed pins; about one or two drops of pus followed the middle one, which was all that was seen; wound looked firm and close; a small amount of lint just over the pedicle, very adherent, which was left. I thought we would use an enema this evening, but she spoke of fatigue after the dressing, as it was somewhat tedious, the lint having become dry; and it was thought better to omit it until to-morrow evening.

1.30 p.m.—Pulse 86; resting quietly, inclined to sleep. Has not passed urine since sometime in the night; feels no uneasiness from it. She has used ice constantly when she wished to moisten her mouth, either in lumps or in water. Directed gradual increase of diet, nothing solid, however, for a day or two yet. She has d ne so well they have thought it unnecessary for me to continue my visits, but will inform me at once should anything untoward occur; they will also keep me informed, at least each alternate day, of her progress, so that I may be able to continue my report to you.

---

Monmouth, Friday Evening, June 8th, 1860.

Dear. Dr.:

On yesterday evening I received the notes of the two preceding days from Dr. Talliaferro, which I subjoin, and not altogether liking their tenor, I determined on visiting the patient to-day, and seeing for myself. I therefore postponed writing until the present. I have just returned, and found *all well*, and an *almost certainty* of speedy recovery. I continue the history from last date.

June 5th. 9, p.m.—Pulse 87; has not recovered altogether from fatigue of dressing wound: otherwise about the same.

6th. 7, a.m. Pulse 92; rested only tolerable through the night; says she does not feel so well; has taken two injections, one of water, the other salt and water. No motion. No thirst or heat of surface. Appetite about the same. Ordered an enema every two and a half hours. 7, p.m.—Pulse 92; no motion of bowels; has taken four injections; otherwise about the same. Oil, half table-spoonful every two and a half hours to two doses, to be followed by enema. Complains of an unpleasant feeling across wound, no tenderness.

7th. 7, a.m.—This morning, pulse 87; more cheerful than yesterday. No action of bowels. The unpleasant sensation has left. Appetite pretty good. Appearance of wound about the same; lint still fast over the pedicle: some unpleasant odor; applied charcoal. Will repeat the oil and enema. Takes no opium. Upon receipt of this, I sent by the same messenger a note advising no particular hurry in moving the bowels, and that I would visit her. By referring to my last note, you will observe that there had been no evacuation of urine for some 10 or 12 hours previous, and as there was nothing said respecting it in this last report of Dr. T., taken in connection with the "unpleasant feeling" and no rest, made me somewhat uneasy concerning her.

I saw her about 10, a.m.—Locked cheerful, entered into conversation, laughed and said she was coming up to town on commencement day, which is in about three weeks, to see her classmates graduate. Her bowels had been moved sufficiently this morning. Appetite not craving, but sufficient. No pain or soreness of bowels or abdomen, and no pain or uneasiness when they were moved. Wound looked firm; the lint covering the pedicle removed. Small suppurating surface about ¾ inch diameter; only pus enough to wet the lint. Removed one of the metallic sutures—the one in connection with the pedicle—the others to be removed in a day or two; pulse 90. Says she slept well last night and feels refreshed and stronger. Voids urine naturally and in sufficient quantity. The depressed condition following the dressing of the wound on the 5th, I now attribute in great measure to the fact that the opium had been discontinued from midnight of the 4th, and as there was a considerable amount of debility from the hemorrhage and strict diet, she was in need of a stimulant. Her natural powers, however, triumphed without artificial means.

Directed a gradual increase of diet; has taken some rice today, which her mother tells me has been the nearest approach to solid food yet.

It does appear to me now that she may be considered out of danger. The wound may be considered healed, as there is but a small point uncovered, and that looking healthy; general appearance and condition of the bowels natural, they having been safely moved. She feels well, and save some unnatural paleness, looks well. Her father thinks the only danger now is in being too confident, and becoming careless in feeding and nursing. This is a good idea, and you see emanates from the proper point, so that we may have less fear of improprieties.

What will be the future effect of the retention of the pedicle in the external wound?

This question has been asked me by Dr. Hamilton, and also by her father, in reference particularly to future pregnancy. Having great faith in the *distensibility* and *elasticity* of *female tissue* in particular, I have answered that it would not prove any obstacle. What say you?

---

Monmouth, June 13th, 1860.

Dear Dr.:

Yours of the 11th is at hand, and as I promised to write you again early this week, I now comply.

I did not hear anything from our patient since my last note until yesterday, when her father came to town. His report was merely a general statement of her condition, which was entirely satisfactory.

He said she was steadily improving; gaining strength as fast as could be expected. Her appetite was good; slept well, and thought she would sit up (in bed at least) the latter part of this week. There was still some very slight discharge from the small surface of the peduncle not yet cicatrized, and from a small spot at the inferior angle of the wound.

Thus far not one unfavorable symptom has arisen, and she says so far as sensation is concerned, she is scarcely aware she has received a wound.

---

Monmouth, July 13th, 1860.

Dear Sir:

Our patient, Miss Eldridge, made her promise good of attending College commencement, which took place on the 5th. She spent the 4th in town, but did not go out to the exercises, reserving herself for the next day. She says she is as well as ever she was; feels fine and lively.

I endeavored to ascertain something of the early history of the case, but failed entirely, as she was scarcely aware of the time she first noticed it. As near as I could learn from her, some eighteen months or two years ago, she thought her abdomen a little large; never noticed any distinct tumor at any time; always felt well, and was not concerned about herself. She only began to think of her condition about two months before you saw her, and then only because she was conscious of more rapid enlargement, which increased up to the time of operating. She says she feels no difference now, but is aware that she is much lessened in size, judging from her clothes. Still she avers that she never felt any particular inconvenience from the tumor, no heaviness, impeded respiration, or anything of that kind.

The distance from Roseville to Monmouth, where Miss E. spent her 4th of July, is 12 miles.

---

I wish to make a very few remarks as to the mode of performing the operation.

It wil be seen that there was no necessity of introducing the hand, or even the fingers, inside the peritoneal cavity. The whole mass was drawn through the wound by pulling upon the collapsed cists, and there being no effusion of blood, or in fact anything else within the abdomen, the membrane was subjected to no rude bandling, or other irritation, except what may have been produced by the entrance of air; and this was quite free, unavoidably. By drawing the stump through the wound, and placing the margin outside the abdominal cavity, the hemorrhage which often occurs, and did in this case from it, was external. The silver pins and wire produced as little irritation as anything else could. Although Dr. Young in one of his notes expressed regret that we had not ligated the stump, I would certainly not do so were I to operate again. The securing the stump in the wound made it impossible for the small sloughs which are always thrown off from it to irritate the peritoneum. It also, had we tied or clamped it, enabled us to avoid the inclusion of the ligature within the cavity. The ecrasseur is better for separating the tumor than a ligature, because, if the latter is used, the peritoneum must be strangulated and inflamed necessarily, and as inflammation spreads along this membrane with great facility, it may invade the abdominal cavity, and thus light up a fatal peritonitis. The hemorrhage that did occur, had it been suspected, could doubtless have been easily checked, and a little vigilance, I think, would render this operative proceedure as safe as such an operation in the nature of things can be.

